# Hypoxic Conditions in Crown Galls Induce Plant Anaerobic Responses That Support Tumor Proliferation

**DOI:** 10.3389/fpls.2019.00056

**Published:** 2019-02-05

**Authors:** Lucy Kerpen, Luca Niccolini, Francesco Licausi, Joost T. van Dongen, Daan A. Weits

**Affiliations:** ^1^Institute of Biology I, RWTH Aachen University, Aachen, Germany; ^2^Department of Biology, University of Pisa, Pisa, Italy; ^3^Scuola Superiore Sant’Anna, Institute of Life Sciences, Pisa, Italy

**Keywords:** crown gall disease, *Agrobacterium tumefaciens*, *Rhizobium radiobacter*, hypoxia, oxygen sensing, N-end rule pathway, ERF VII

## Abstract

*Agrobacterium tumefaciens* infection of wounded plant tissues causes the formation of crown gall tumors. Upon infection, genes encoded on the *A. tumefaciens* tumor inducing plasmid are integrated in the plant genome to induce the biosynthesis of auxin and cytokinin, leading to uncontrolled cell division. Additional sequences present on the bacterial T-DNA encode for opine biosynthesis genes, which induce the production of opines that act as a unique carbon and nitrogen source for *Agrobacterium*. Crown galls therefore become a very strong sink for photosynthate. Here we found that the increased metabolic demand in crown galls causes an increase in oxygen consumption rate, which leads to a steep drop in the internal oxygen concentration. Consistent with this, plant hypoxia-responsive genes were found to be significantly upregulated in crown galls compared to uninfected stem tissue. Following this observation, we aimed at understanding whether the low-oxygen response pathway, mediated by group VII ethylene response factor (ERF-VII) transcription factors, plays a role in the development of crown galls. We found that quintuple knock-out mutants of all ERF-VII members, which are incapable of inducing the hypoxic response, show reduced crown gall symptoms. Conversely, mutant genotypes characterized by constitutively high levels of hypoxia-associated transcripts, displayed more severe crown gall symptoms. Based on these results, we concluded that uncontrolled cell proliferation of crown galls established hypoxic conditions, thereby requiring adequate anaerobic responses of the plant tissue to support tumor growth.

## Introduction

Crown gall disease is caused by the pathogenic bacterium *Agrobacterium tumefaciens* (Escobar and Dandekar, 2003), which, after a revised phylogenetic analysis, was attributed among the *Rhizobium* genus as *Rhizobium radiobacter* (Young et al., 2001). Crown gall formation is the result of uncontrolled proliferation of neoplastic tissue that is genetically reprogrammed by the insertion of bacterium-encoded DNA fragments to produce opines that *Agrobacterium* feeds on. Virulent *Agrobacteria* contain a tumor inducing plasmid (pTi) that carries the sequences that are transferred to the host plant (transfer-DNA, T-DNA), containing genes required for their conjugation, cell proliferation, opine catabolism genes, and virulence loci. The latter mediates the broad host range of the *Agrobacterium* (Thomashow et al., 1980; Gelvin, 2003), and this feature has been widely employed as a tool in biotechnology for the generation of transgenic plants (De Cleene and De Ley, 1976; Păcurar et al., 2011). Instead, the agricultural impact of crown gall disease is limited to a subset of plant species, which include nut trees, perennial fruit trees, vines, and some ornamental plants such as rose (Escobar et al., 2002; Aysan and Sahin, 2003; Chen et al., 2007). In these species, crown gall disease causes significant yield loss due to re-allocation of nutrients to the metabolically active crown galls and a constriction of the vasculature tissue, which limits xylem and phloem transport to the organs above (Gohlke and Deeken, 2014).

Upon infection, *A. tumefaciens* integrates its T-DNA into the plant genome, likely through hijacking of the endogenous plant nuclear transport and DNA repair mechanisms (Chilton et al., 1977; Tzfira and Citovsky, 2006). The T-DNA contains two major functional sets of genes, namely opine metabolism genes and oncogenes. The latter induces the biosynthesis of plant hormones auxin and cytokinin, while other oncogenes may also increase the sensitivity of the plant tissue to these hormones (Britton et al., 2008). The upregulated auxin and cytokinin levels promotes uncontrolled and rapid cell division, leading to the production of tumor-like heterotrophic galls (Gohlke and Deeken, 2014). A second class of genes induce the biosynthesis of carbohydrate and amino acid derived opines, which act as unique energy source for the *Agrobacteria* present in the gall (Guyon et al., 1980). Opine catabolism genes are also located on the Ti plasmid, but are not integrated into the host genome, thus restricting the use of opines as an energy source to *Agrobacteria* only.

The rapid growth of crown gall tumors induces specific changes in gene expression and metabolic pathways (Deeken et al., 2006). Interestingly, the expression of genes involved in ethanol fermentation, *alcohol dehydrogenase 1* (*ADH1*) and *pyruvate decarboxylase 1* (*PDC1*), were found to be upregulated in crown gall tissue, as compared to uninfected wounded stem tissue (Deeken et al., 2006), hinting at potentially underlying hypoxic conditions within the crown galls. In plants, the expression of hypoxia responsive genes is regulated by the Arg-Cys N-end rule pathway (Gibbs et al., 2011; Licausi et al., 2011; Weits et al., 2014). This pathway regulates the O_2_, and NO-dependent proteolysis of group VII ethylene response factors (ERF-VII) (Licausi et al., 2011; Bailey-Serres et al., 2012; Gibbs et al., 2014). In this process, the plant cysteine oxidases (PCO) oxidize the N-terminal cysteine of the ERF-VII transcription factors, after the co-translational removal of N-terminal methionine by methionine aminopeptidases (Bradshaw et al., 1998; Weits et al., 2014; White et al., 2017). The oxidized cysteine is recognized by arginine transferases (ATE) which conjugate an arginine residue to the N-terminus, thus providing a specific substrate for the E3 ligase PROTEOLYSIS 6 (PRT6) (Garzón et al., 2007; Holman et al., 2009). PRT6 adds ubiquitin units to the ERF-VII transcription factors so that the polyubiquitinated protein is subsequently degraded via the 26S proteasome (Gibbs et al., 2011; Licausi et al., 2011; Varshavsky, 2011). Therefore, ERF-VII are stabilized under hypoxic conditions and activate the anaerobic response.

Adaptation to hypoxic stress in plants comprises the upregulation of several genes (Mustroph et al., 2010), which include those involved in fueling glycolysis via degradation of sucrose reserves by sucrose synthases (SUS) (Zeng et al., 1999). Additional reactions activated as part of the adaptive program to hypoxic conditions involve regeneration of NADH through the induction of ethanol fermentation, and this is catalyzed by the enzymes PDC and ADH (Ismond et al., 2003). Nitric oxide accumulation is prevented by class 1 PHYTOGLOBIN 1 (AtPgb1), with the consequent production of NO^3−^ (Igamberdiev et al., 2005). Finally, a group of genes is induced that control and attenuate low oxygen signaling termed *hypoxia response attenuator 1* (*HRA1*) (Giuntoli et al., 2014), and *PCO* (Weits et al., 2014). The role of several hypoxia-inducible genes remains undiscovered, such as *LOB DOMAIN-CONTAINING PROTEIN 41* (*LBD41*) and several *hypoxia-responsive unknown protein* (*HUP*) genes (Mustroph et al., 2010).

We hypothesized that high rates of cellular proliferation and opine production causes hypoxia in crown galls, which in turn triggers plant hypoxic responses, including fermentation. In this study, we used oxygen microsensors and hypoxia-responsive reporters to determine if crown gall tumors induced by *A. tumfaciens* are hypoxic. Furthermore, we analyzed if the resulting hypoxic responses induced by the plant contributes to the proliferation of crown gall disease.

## Materials and Methods

### *Agrobacterium* Infection

For *A. tumefaciens* infection studies, seeds of *Arabidopsis thaliana* were sown in moist soil and stratified at 4°C for 48 h. Seeds germinated at 20°C in an 18 h light and 6 h dark photoperiod. To mediate *A. tumefaciens* infection, a 5 mm incision was made on young inflorescence stalk tissue using a razorblade. For induction of crown gall disease, *A. tumefaciens* strain 30205 (Leibniz-Institut DSMZ – Deutsche Sammlung von Mikroorganismen und Zellkulturen GmbH) was applied to the wounded inflorescence stalk. Prior to induction, *A. tumefaciens* was grown overnight in liquid medium to an OD of 1.0. Liquid medium contained 5 g/l peptone and 3 g/l meat extract, pH 7.0. Following inoculation, infected plants were brought back to the growth conditions described above.

### Analysis of Crown Gall Symptoms

For evaluation of disease symptoms, micrographs were taken 21 days post infection (dpi) using a Leica M205 FA stereomicroscope equipped with a Leica DFC450 C camera. To determine the mass of each gall, tumors were carefully removed from the stem using a razorblade to minimize contamination of stem tissue and weight on a precision balance (Sartorius AZ124). Experiments were repeated at least 4 times for each genotype.

### GUS-Staining

For histochemical GUS-staining, plants of *A. thaliana*, harboring a construct consisting of the promoter of the *PCO1* gene or the fivefold Hypoxia Responsive Promoter Element (*HRPEx5*) (Mustroph et al., 2010) fused to the GUS reporter gene were grown and inoculated with *A. tumefaciens* as described above (Supplementary Table S1). The segment of the stem harboring crown gall tumors was harvested 7, 14, and 28 dpi. Following harvesting, the segments were fixed in ice cold acetone (90%) for 1 h, and subsequently immersed in GUS-staining solution (Jefferson et al., 1987). The samples were vacuum infiltrated briefly to promote distribution of the staining solution in to the plant tissue. GUS-staining was performed at 37°C for 5–12 h and stopped by exchanging the staining solution for 70% ethanol. Further destaining of the samples was achieved using several changes of 70% ethanol.

### GFP-Imaging

For GFP-imaging, *A. thaliana* plants, in which the promoter of the *PCO1* gene was fused to a GUS-GFP reporter, were grown and inoculated with *A. tumefaciens* as described above. Crown galls were analyzed using a Leica M205 FA stereomicroscope equipped with a Leica DFC450 C camera at three different developmental stages (7, 14, and 28 dpi). GFP was detected using a band pass ET GFP Filter set (Leica).

### qRT-PCR

For gene expression studies, crown galls were harvested 21 dpi and material of three tumors was combined to make up each biological replicate. As control, stems were inoculated with a non-virulent *A. tumefaciens* strain 30147 (Leibniz-Institut DSMZ - Deutsche Sammlung von Mikroorganismen und Zellkulturen GmbH). Total mRNA extraction was carried out as previously described (Kosmacz et al., 2015). The mRNA-concentration was measured by Nanodrop. 2750 μg of mRNA template was used for TURBO DNAse (Thermo-Fisher scientific) treatment according to the manufactures instructions and complementary DNA synthesis was carried out using the RevertAid First Strand cDNA Synthesis Kit (ThermoFisher scientific). PowerUp^TM^ SYBR^TM^ Green Master Mix was used for qRT-PCR. *UBIQUITIN10* was used as reference gene, after confirming that its expression is unaltered by *A. tumefaciens* infection. Relative gene expression was calculated according to the ΔΔCt method (Livak and Schmittgen, 2001). A full list of primers used in this study is provided in Supplementary Table S2.

### Oxygen Measurements Using Microsensors

The oxygen concentration in live crown galls and callus was measured at 14 and 28 dpi using a FireStingO_2_ oxygen meter and a retractable needle-type OXR50 oxygen microsensor with a tip diameter of 50 μm (Pyroscience). Prior to the measurements, calibration was performed using pure N_2_ gas and atmospheric air. At least four measurements were performed for each tissue, to calculate the average internal oxygen concentration. Stems of non-inoculated plants were used for comparison.

### Oxygen Consumption Measurements

Crown galls were carefully removed from infected *A. thaliana* plants using a razorblade. Uninfected stems were harvested as control. The fresh weight of plant material was determined precisely prior to the O_2_ measurements, on a precision balance (Sartorius AZ124). On average, each replicate had a mass of approximately 25 mg. Next, O_2_ consumption was measured with an integrated optical oxygen sensor in respiration vials (Pyroscience) filled with sterile distilled H_2_O and under continuous mixing using a magnetic stirrer. The oxygen consumption rate was calculated by dividing the decrease in oxygen concentration by the time and corrected for the weight of each sample.

### Transient *Agrobacterium* Transformation

Infiltration of *A. thaliana* leaves with *A. tumefaciens* was performed as previously described with some modifications (Zipfel et al., 2006). Disarmed *Agrobacterium* GV3101 strains carrying a *Renilla*-intron luciferase construct (Cazzonelli and Velten, 2003) were grown overnight in LB medium and then collected by centrifugation and resuspended in 2.5 volume of AB-MES (Wu et al., 2014), containing 200 μM acetosyringone and antibiotics (Rifampicin 50 mg/L, Gentamicin 50 mg/L, Kanamycin 50 mg/L). Following growth for 5–6 h, cells were resuspended in AB-MES supplemented with 200 μM acetosyringone at an OD of 0.5 and injected into leaves of 4-week old plants. Six plants were used per genotype and 3 leaves were infiltrated per plant. Leaves were harvested 3 dpi and luciferase activity was quantified according to the manufactures instructions (Promega).

### Callus Induction and Imaging

Callus induction was carried out using root explants of 7-day old *promPCO1:GG* and *promPCO2:GG* plants on callus induction medium according to (Sugimoto et al., 2010). GFP fluorescence of calli was imaged using an inverted Zeiss LSM 800 confocal laser scanning microscope, equipped with a WPlan-Apochromat40x/1,0DICVIS-IRM27 dipping objective. N-(3-Triethylammoniumpropyl)-4-[6-[4-(Diethylamino) Phenyl] Hexatrienyl] Pyridinium Dibromide staining (FM4-64 dye, Thermo Fisher Scientific) was carried out according to the manufacturer’s instructions.

### Statistical Analysis

To evaluate the differences between genotypes and tissue inoculated with *A. tumefaciens* one-way analysis of variance (ANOVA) tests were performed using the SigmaPlot 14.0 software (Systat Software). After one-way ANOVA, a Holm-Sidak *post hoc* test was performed to assess if there is a statistically significant difference between the measurements. Asterisks indicate a significant difference between the genotypes or tissue (^∗^<0.05, ^∗∗^<0.01). BoxPlotR (Spitzer et al., 2014) was used to generate boxplots. Histograms show mean values ± standard deviation (SD).

## Results

### *Agrobacterium tumefaciens* Induced Crown Gall Tumors Are Hypoxic

**FIGURE 1:**
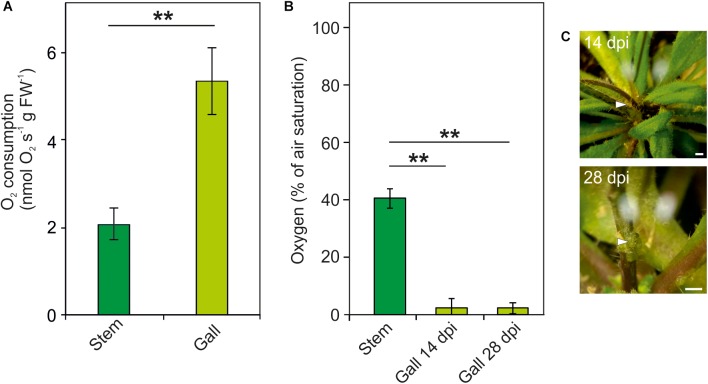
Increased oxygen consumption causes hypoxia in crown galls. **(A)** Oxygen consumption rate of crown galls and stem tissue. O_2_ consumption rate over time was calculated per gram fresh weight. **(B)** Oxygen concentration (% of air saturation) in crown galls, measured at 14 and 28 dpi using an oxygen microsensor. **(C)** Photographs of tumor formation on *Arabidopsis* stems after 14 and 28 days of inoculation with *Agrobacterium tumefaciens*. Arrows indicate the position of the microsensor. Scale bars, 1 mm. Data are presented as means ± SE of at least 4 independent measurements. Stars indicate a statistically significant difference [^∗^<0.05, ^∗∗^<0.01, one-way analysis of variance (ANOVA) followed by Holm-Sidak *post hoc*-test].

Induction and proliferation of crown galls due to *Agrobacterium* infection leads to an increased energy demand. We therefore measured oxygen respiratory rates in freshly isolated crown galls and uninfected plant tissue. We observed a more than 2.5 times increase of the oxygen consumption rate in crown gall tissue, as compared to non-inoculated stem tissue (Figure 1A). Subsequently, we measured the oxygen concentration within intact crown galls using oxygen microsensors at 14 and 28 dpi. In non-inoculated stem tissues, an oxygen gradient was observed, which reaches its lowest concentration of on average 40% of air saturation in the core of the stem (Figure 1B). In contrast, in crown gall tumor tissue the O_2_ concentration dropped to much lower values (<5% of air saturation) at both developmental stages (Figure 1B,C). Apparently, the increased oxygen consumption rate of the crown gall tumors induces a much steeper oxygen gradient, as compared to the oxygen gradient in non-infected tissue.

**FIGURE 2:**
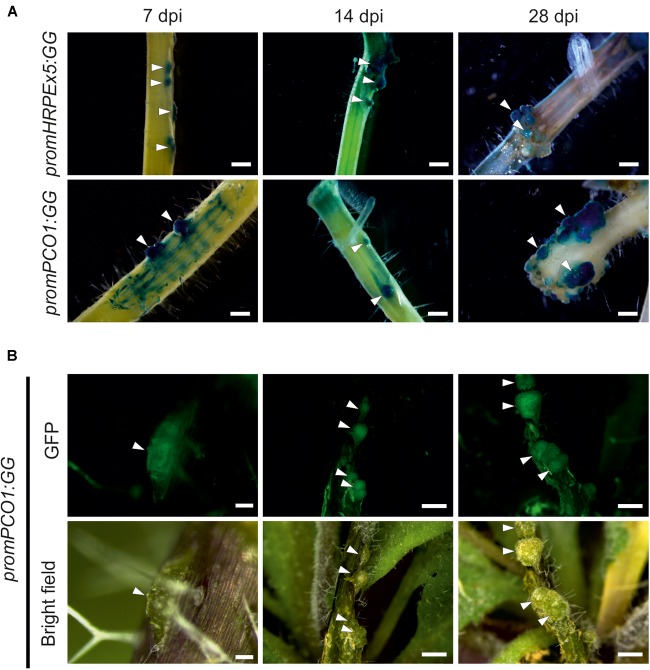
The low oxygen reporter gene *PCO1* and the hypoxia inducible promoter *HRPEx5* are specifically induced in crown gall tumors at different development stages. **(A)** Histochemical GUS staining of *promPCO1:GG* and *promHRPEx5:GG* infected with *A. tumefaciens*. Staining was performed at 7, 14, and 28 dpi. Arrows indicate crown gall formation on the stem. **(B)** GFP fluorescence and bright field images of crown galls, harboring the *promPCO1:GG* construct. GFP imaging was performed at 7, 14, and 28 dpi. Scale bars indicate 100 μm for *promPCO1:GG* at 7 dpi, all other scale bars indicate 1 mm.

Next, we confirmed the O_2_ measurements of the sensors using hypoxia responsive promoter reporters, which provide a readout for underlying hypoxic conditions in the galls. These also allowed us to visualize activation of hypoxia in the earlier time points in crown gall development, where the O_2_ microsensors were too large to be used. We employed genetically encoded hypoxia signaling reporters based on the promoter of the *PCO1* gene, which is strongly and specifically induced upon hypoxia (Weits et al., 2014), fused to a chimeric β-glucuronidase (GUS) – enhanced green fluorescent protein (eGFP) reporter (*promPCO1:GG*). Additionally, we utilized a synthetic promoter composed of a fivefold repeat of the Hypoxia Responsive Promoter Element that was also linked to the combined GUS-GFP reporter cassette (*promHRPEx5-GG*). The *HRPE* is an evolutionary conserved 12-bp cis-regulatory motif, that provides hypoxia-inducibility in *A. thaliana* (Gasch et al., 2015). Both hypoxia reporters were previously shown to be strongly activated upon low oxygen treatments (Weits et al., 2014; Gasch et al., 2015). Next, we infected transgenic plants harboring these constructs, and performed GUS staining and GFP imaging at several stages post infection (7, 14, and 28 dpi). We observed the first crown gall symptoms at 7 dpi, which already showed strong GUS staining of the *promPCO1:GG* and *promHRPEx5-GG* low oxygen reporters, while the surrounding stem tissue showed only a weak or no activation of the reporters (Figure 2A). Both hypoxia reporters remained strongly activated at the 14 and 28 dpi time points. GFP fluorescence of *promPCO1:GG* lines displayed a comparable specific activation of the hypoxia reporter in crown galls, during all time points, and no GFP signal was observed in stem tissue (Figure 2B). These observations show that crown galls become hypoxic during their formation and remain hypoxic throughout their development.

### Plant Anaerobic Genes Are Induced in Crown Galls

**FIGURE 3:**
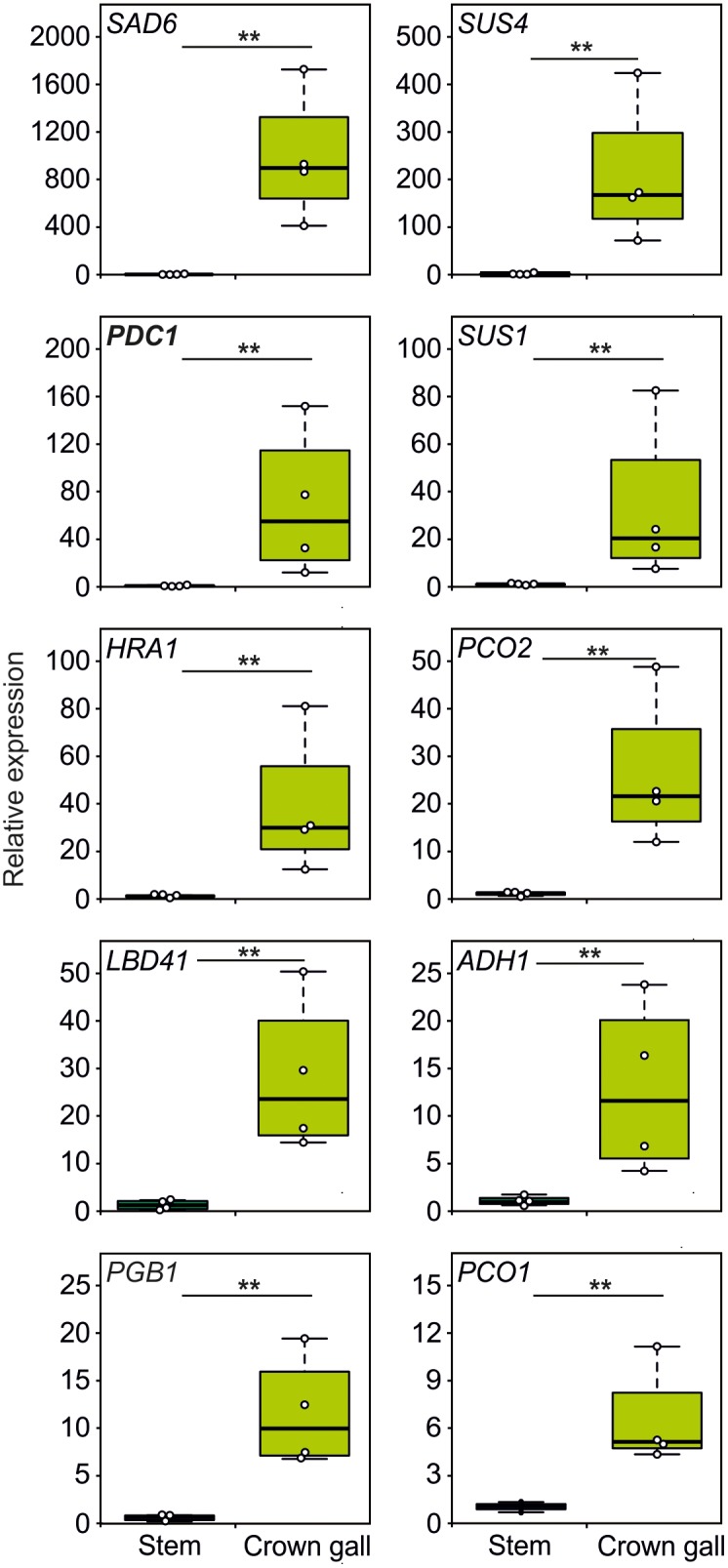
The expression of hypoxia related genes is upregulated in crown gall tumors. Relative expression of hypoxia-responsive genes in crown gall tumors compared with stem tissue inoculated with a non-tumor producing *Agrobacterium* strain. Data are presented as boxpots. Stars indicate a statistically significant difference [^∗^<0.05, ^∗∗^<0.01, ANOVA followed by Holm-Sidak *post hoc* test].

Since crown galls are hypoxic, we investigated if plant transcripts associated with tolerance to hypoxic stress are also induced in galls. Interestingly, we found that all 10 tested genes, belonging to the core set of hypoxia-inducible transcripts (Mustroph et al., 2010), were strongly upregulated in crown galls, as compared to stem tissue that was infected with a non-virulent *A. tumefaciens* strain (Figure 3 and Supplementary Table S3). Taken together, crown galls clearly show an upregulation of the majority of measured hypoxia associated transcripts.

### The Plant Hypoxic Response Supports Tumor Development

**FIGURE 4:**
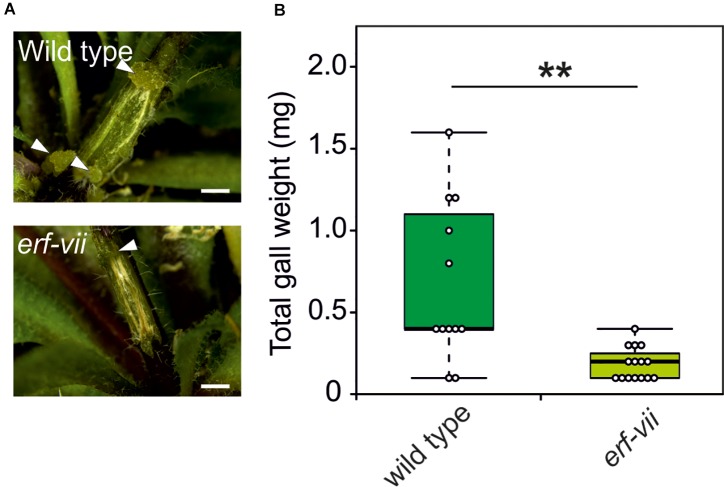
*Arabidopsis* lines with a constitutively repressed hypoxia response show reduced crown gall symptoms. **(A)** Representative images of crown gall disease symptoms at 21 dpi in *erf-vii* and wild type plants. Scale bar, 1 mm. Crown galls are indicated with white arrows. **(B)** Gall fresh weight of *erf-vii* and wild type plants at 21 dpi. Data are presented as boxplots. Stars indicate a statistically significant difference [^∗^<0.05, ^∗∗^<0.01, ANOVA followed by Holm-Sidak *post hoc* test].

Based on the increased expression of hypoxia-responsive genes in crown gall tumors, we hypothesized that the anaerobic response of the plant contributes to the development and maintenance of crown gall tissue. In plants, adaptation to hypoxic conditions is mediated by the induction of anaerobic genes under the control of ERF-VII transcription factors. To investigate if ERF-VIIs are required for the development of crown galls, we analyzed crown gall symptoms in *erf-vii* quintuple mutants (Abbas et al., 2015) infected with *A. tumefaciens*. Indeed, crown gall symptoms were decreased in the *erf-vii* knockout lines (Figure 4A), which also corresponded with a significantly decrease in total crown gall weight (Figure 4B). These results demonstrate that the hypoxic response mediated by ERF-VII contributes to the development of crown gall tumors.

**FIGURE 5:**
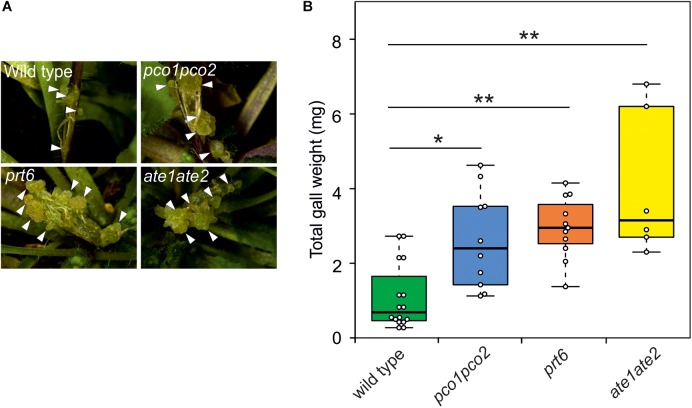
Arabidopsis lines with a constitutively active hypoxia response show reduced crown gall symptoms. **(A)** representative images of crown gall disease symptoms at 21 dpi in wild type, *pco1pco2*, *prt6*, and *ate1ate2* plants. Scale bar, 1 mm. Crown galls are indicated with white arrows. **(B)** Gall fresh weight of wild type, *pco1pco2*, *prt6* and *ate1ate2* plants at 21 dpi. Data are presented as boxplots. Stars indicate a statistically significant difference [^∗^<0.05, ^∗∗^<0.01, ANOVA followed by Holm-Sidak *post hoc* test].

To investigate whether induction of the hypoxic response indeed positively affects the development of crown galls, we analyzed crown galls symptoms in several previously described N-end rule mutants (*pco1pco2*, *prt6*, and *ate1ate2*) (Garzón et al., 2007; Holman et al., 2009; Weits et al., 2014). These mutants lack activity of essential components of the Arg-Cys branch of the N-end rule pathway, which leads to an accumulation of ERF-VII proteins that promote the induction of hypoxia responsive genes (Gibbs et al., 2011; Licausi et al., 2011). Indeed, we observed a significant increase in gall symptoms and gall weight after 21 dpi in *pco1pco2*, *prt6* and *ate1ate2* mutants, indicating that the induction of the anaerobic response promotes crown gall development (Figure 5A,B).

### The N-End Rule Pathway Does Not Affect the Susceptibility of Plants to *A. tumefaciens* Mediated Transformation

**FIGURE 6:**
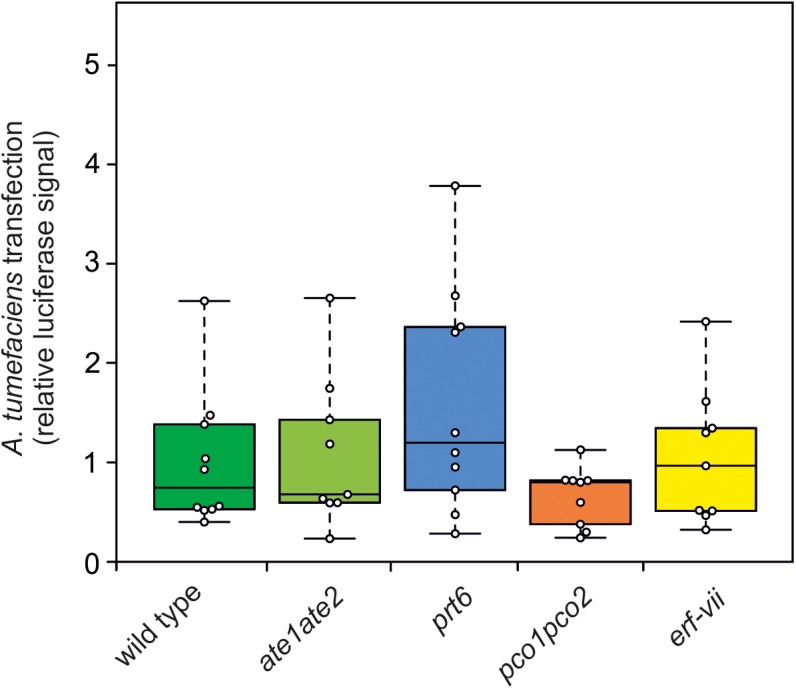
N-end rule mutants do not show altered susceptibility to *A. tumefaciens* infection. Quantification of the relative luminescence of leaves from wild type, *ate1ate2*, *prt6*, *pco1pco2*, and *erf-vii* lines infiltrated with a disarmed GV3101 *A. tumefaciens* strain carrying a *Renilla* luciferase reporter. The *Renilla* luciferase gene carries an intron to restrict its expression in *Agrobacterium*. Relative luciferase signal of each genotype was calculated per μg of protein. ANOVA was performed to test for statistically significant difference between the genotypes.

Previously it has been reported that the N-end rule plays a role in the immune response to a wide range of pathogens, including bacteria (de Marchi et al., 2016; Vicente et al., 2018). Therefore, we set out to investigate to which extent altered susceptibility of N-end rule mutants to *A. tumefaciens* infection may play a role in the development of crown gall tumors. For this purpose, we used a disarmed GV3101 *A. tumefaciens* strain to entangle the potential role that the N-end rule plays during the initial stage of *Agrobacterium* infection and transfection, from its role in regulating the plant anaerobic response in hypoxic galls. The susceptibility of *ate1ate2*, *prt6*, *pco1pco2* and *erf-vii* mutants was assessed by infiltration of an *Agrobacterium* carrying a *Renilla* reporter gene in its T-DNA, whose expression is limited to eukaryotic cells by the presence of an intron from the castor bean catalase gene *CAT-1* (Cazzonelli and Velten, 2003). Compared to the wild type, none of the mutants showed a significantly different luminescence signal after 3 days of infection, indicating that they are not affected in their susceptibility to *Agrobacterium* infection (Figure 6).

### Callus Regeneration Induces the Plant Anaerobic Response and Is Associated With a Low Oxygen Concentration

**FIGURE 7:**
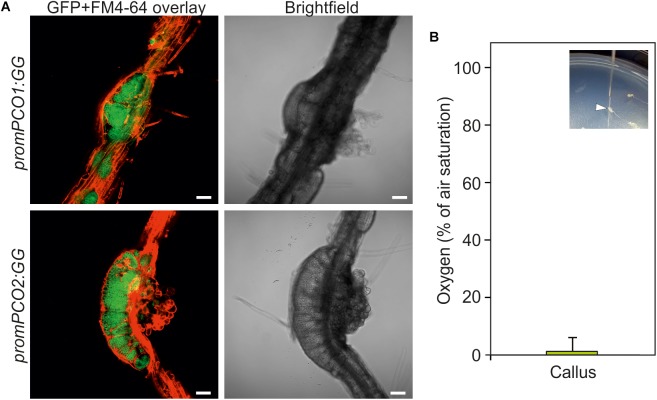
Callus is hypoxic. **(A)** GUS activity of *promPCO1:GG* and *promPCO2:GG* hypoxia-signaling reporters in root-derived calli. GFP fluorescence of *promPCO1:GG* and *promPCO2:GG* in calli. FM4-64 was used to stain the cell membranes. Scale bars, 50 μm. **(B)** Direct oxygen measurements in callus using an oxygen microsensor (% of air saturation). The inlet shows insertion of the microsensor into a callus.

The establishment of hypoxia in crown gall tumors raised the question if this a unique feature associated with crown gall tumor proliferation, or if it may occur more commonly in actively dividing and tightly packed tissue. With the aim of investigating this, we analyzed the oxygen availability in callus, which is characterized by a rapid proliferation of undifferentiated cells from pericycle tissue in the presence of auxin and cytokinin (Atta et al., 2009). The hypoxia signaling reporters *promPCO1:GG* and *promPCO2:GG* each showed a strong induction in root-derived calli as indicated by intense GFP fluorescence, as compared to the weak expression of these reporters in the neighboring root tissue (Figure 7A). Moreover, direct oxygen measurements in the callus showed that this tissue is indeed hypoxic, suggesting that hypoxic conditions may be a common feature of rapidly dividing tissue (Figure 7B).

## Discussion

Upon infection of plant tissue by the bacterium *A. tumefaciens*, plant cells are genetically transformed with the genes present on the T-DNA to induce rapidly expanding tumor tissue, from which the bacteria are nourished. As a consequence, both opine metabolism and proliferation of plant cells are strongly activated, which imposes a strong demand on the plant’s energy metabolism. Therefore, the crown galls become a strong metabolic sink and vasculature formation is induced to provide additional nutrients to the tumors (Melnyk, 2017). Previous reports identified the induction of gene expression of the fermentative enzymes *PDC1* and *ADH1* in crown galls (Deeken et al., 2006), but it was not shown if this response was linked to changes in the concentration of oxygen inside the tumor tissue. Using both oxygen microsensors and genetically encoded hypoxia reporters, we now show that crown gall tumors are indeed hypoxic (Figure 1). Interestingly, these hypoxic conditions are already established early after infection (7 dpi), when tumors have not expanded markedly (Figure 2). Apparently, the steep oxygen gradient in the crown galls is not a direct consequence of the occurrence of bulky tumor tissue, but rather the result of increased oxygen consumption rate by the infected tissue.

To verify that the steep oxygen gradient that we measured in crown gall tumors is indeed caused by the upregulation of respiratory activity, we measured the oxygen consumption rate of this tissue (Figure 1A). We found that galls have an increased oxygen consumption rate compared to stem tissue, which likely explains the hypoxic conditions inside the galls. We therefore concluded that the increased energy demand that is required by the plant cells to maintain tumor cell proliferation and opine metabolism, induces a steep oxygen gradient in the tumor tissue. This observation shows a striking resemblance to solid animal cancer tumor tissue, which is also characterized by very high metabolic activity that is linked to increased oxygen consumption rates and concomitant hypoxia (Vaupel and Harrison, 2004; Schito and Semenza, 2016).

The low oxygen concentrations within the gall tumor tissue induced the expression of all 10 tested genes that are known to be responsive to hypoxia (Figure 3). The expression of the genes *SUS1* and *SUS4*, encoding for sucrose synthase enzymes, were induced upon *A. tumefaciens* infection. Both *SUS1* and *SUS4* were previously found to be strongly upregulated in crown galls (Deeken et al., 2006). Upregulation of SUS is a well-known adaptive response to low oxygen stress in plants, as the cleavage of sucrose to glucose and fructose by SUS is energetically more favorable, then when the hydrolysis of sucrose is catalyzed by invertases (Bologa et al., 2003). The upregulation of the *ADH1* and *PDC1* genes that encode for the fermentative enzymes alcohol dehydrogenase and pyruvate decarboxylase are also part of an important adaptive metabolic response of the plant to low oxygen conditions (Figure 3, Gohlke and Deeken, 2014). Clearly, the upregulation of these genes in *Arabidopsis* tumor tissue are linked to a plant adaptive response to hypoxic conditions inside the gall.

On first sight, it might seem counterintuitive that gall tumors have high respiratory activity, while their internal oxygen concentration is very low (Figure 1). Despite its low concentration, oxygen is apparently not a limiting substrate for oxidative phosphorylation in this tissue as long as the flux of oxygen into the tissue along its concentration gradient remains high. A similar situation has been described in dense cancer tumors that may also maintain respiratory ATP production even in the presence of hypoxic conditions (Kim and Dang, 2006; Gogvadze et al., 2008; Semenza, 2012). Remarkably, while glycolysis and fermentation are strictly activated under hypoxic conditions, cancer tissue can activate anaerobic metabolism while there is sufficient O_2_ for oxidative phosphorylation (Lopez-Lazaro, 2008). Here, glycolysis functions not just to provide additional NADH for respiration, but it is also essential to provide the building blocks for biosynthesis in rapidly dividing tissue. This phenomenon has been well described as the Warburg effect. Moreover, although oxidative phosphorylation is a more efficient process per molecule of glucose, glycolysis can produce ATP significantly faster (Pfeiffer et al., 2001; Bui and Thompson, 2006). Therefore, we suggest that also in crown gall tumors the Warburg effect is activated to keep up with the rapid proliferation rates of this tissue.

Other genes that were found to be upregulated in the crown gall tumors are involved in the signaling cascade by which plants activate hypoxic responses. *PCO1* and *PCO2* encode for the cysteine oxidases that oxidize the N-terminal cysteine in ERF-VII proteins, thereby promoting their destabilization (Weits et al., 2014; White et al., 2017). Thus, PCO1 and PCO2 act to repress hypoxic responses in aerobic conditions, and act as a negative feedback loop to control the strength of these responses under hypoxia. HRA1 regulates the activity of ERF-VII members, and thereby its induction under hypoxia also acts as a negative feedback loop to modulate this response under fluctuating oxygen conditions (Giuntoli et al., 2014). However, the upregulation of *HRA1*, *PCO1* and *PCO2* in crown galls is apparently not sufficient to abolish the plant anaerobic response in this tissue (Figure 3). For PCO this is due to the remarkably low O_2_ concentration in the tumors (<5% air saturation, Figure 1B), which is below the K_m_ of PCO for oxygen (White et al., 2018).

We found that the fatty acid desaturase *SAD6* is induced in crown galls (Figure 3). The genes encoding for the desaturases SAD6 and FAD3 were previously shown to display increased expression in crown galls (Klinkenberg et al., 2014). *SAD6* and *FAD3* are hypoxia-inducible and they were proposed to promote tolerance to hypoxia and drought in crown galls through production of unsaturated fatty acids. While the *fad3-2* mutant developed significantly smaller galls under normal conditions, the down regulation of *SAD6* through RNA interference did not reduce growth of crown galls, hinting at the importance of both genes in the maintenance of crown galls. Taken together, it becomes clear from these data that the plant responds to the infection of *A. tumefaciens* by activating various adaptive responses to hypoxia.

The expression of hypoxia inducible genes is mediated via the hypoxia-dependent stabilization of ERF-VII transcription factors (Gibbs et al., 2011; Licausi et al., 2011). The conditional stabilization of these proteins is controlled by the Arg-Cys N-end rule pathway (Varshavsky, 2011; Tasaki et al., 2012). Genes belonging to the N-end rule pathway may thus provide an interesting target for plant breeders to produce crops resistance to *A. tumefaciens* infection. Therefore, we analyzed what impact modifications to the low-oxygen signaling cascade had on the development of crown gall tumors after *A. tumefaciens* infection. Knocking out all genes encoding for the ERF VII transcription factors, strongly inhibited the development of the crown galls (Figure 4). Apparently, activation of plant adaptive responses to low oxygen is essential for the development of the bacterial-induced tumors, because it allows the plant cells to maintain energy supply for opine production, and cell proliferation. Interestingly, when components of the N-end rule pathway were silenced, the tumors that developed were significantly larger, as compared to when crown galls were induced on wild type plants (Figure 5). Apparently, stabilization of the ERF VII proteins, which was shown previously to trigger the hypoxic responses of plants (Gibbs et al., 2011; Licausi et al., 2011), supports tumor growth.

The upregulation of hypoxia responsive genes has also been observed in clubroot and root-knots (Gravot et al., 2016), suggesting that the activation of hypoxia responsive genes is a common features of pathogen induced tumor formation in plants. Moreover, similar to our findings in crown gall induction, clubroot symptoms were reduced in the *erf-vii* mutant, while they were enhanced in the *prt6* mutant. Therefore, manipulation of the plant anaerobic response, would provide a promising goal for plant breeding approaches that aim to increase resistance against gall inducing pathogens. However, it must be considered that the plant anaerobic response mediated by ERF-VII is also critical for tolerance to submergence stress (Gibbs et al., 2011; Licausi et al., 2011; Riber et al., 2015), so the manipulation of this pathway may result in unwanted side effects.

The N-end rule has been implicated in plant responses to a wide range of pathogens, broadening the role that this pathway plays in plant immunity (de Marchi et al., 2016; Vicente et al., 2018). Based on these findings, altered crown gall symptoms in *prt6*, *ate1ate2* and *erf-vii* could also be explained by the role that the N-end rule plays in plant defense responses. Therefore, we investigated the susceptibility of plants to *Agrobacterium* infection and transfection using a disarmed strain harboring a *Renilla*-intron reporter. None of these mutants showed altered *A. tumefaciens* mediated transformation, indicating that these lines are not affected in their susceptibility to *Agrobacteria* (Figure 6). Instead, we suggest that proteolysis of ERF-VII by the N-end rule play a role in tumor development by regulating the plant anaerobic response to sustain energy production under hypoxic conditions in the galls.

We also observed hypoxic conditions and the concomitant induction of hypoxia-responsive genes in hormone-induced callus (Figure 7), indicating that tissue with rapid cell division rates consumes sufficient oxygen to cause internal low oxygen conditions. This would also imply that hypoxia is a common occurrence in proliferating plant tissue. It remains to be discovered if the role that the N-end rule plays in crown gall development may also extend to other rapidly dividing tissue.

## Conclusion

In this study, we show that *A. tumefaciens* induced crown gall tumors are hypoxic due to their high energy demand and concomitant upregulation of respiratory oxygen consumption. The plant cells respond to these hypoxic conditions by activating adaptive responses to hypoxia that aim to maintain energy production. Further activation of the ERF-VII-mediated hypoxic response pathway resulted in larger tumors, while abolishing the ERF-VII mediated responses significantly reduced the formation of crown gall tumors. Decreasing the hypoxic response of crown gall tumors may be considered as a viable strategy for the breeding of cultivars with higher resistance against *Agrobacterium* infection.

## Author Contributions

DW and JvD planned and supervised the project. FL assisted in the interpretation of the data. LK, DW, and LN conducted the experiments and analyzed the data. DW wrote the manuscript, and JvD and FL critically assessed it. All authors revised the final version of the manuscript.

## Conflict of Interest Statement

The authors declare that the research was conducted in the absence of any commercial or financial relationships that could be construed as a potential conflict of interest.

## References

[B1] Abbas M. Berckhan S. Rooney D. J. Gibbs D. J. Vicente Conde J. Sousa Correia C. (2015). Oxygen sensing coordinates photomorphogenesis to facilitate seedling survival. *Curr. Biol.* 25 1483–1488. 10.1016/j.cub.2015.03.060 25981794 PMC4454774

[B2] Atta R. Laurens L. Boucheron-Dubuisson E. Guivarc’h A. Carnero E. Giraudat-Pautot V. (2009). Pluripotency of arabidopsis xylem pericycle underlies shoot regeneration from root and hypocotyl explants grown in vitro. *Plant J.* 57 626–644. 10.1111/j.1365-313X.2008.03715.x 18980654

[B3] Aysan Y. Sahin F. (2003). An outbreak of crown gall disease on rose caused by *Agrobacterium tumefaciens* in turkey. *Plant Pathol.* 52 780–780. 10.1111/j.1365-3059.2003.00889.x

[B4] Bailey-Serres J. Fukao T. Gibbs D. J. Holdsworth M. J. Lee S. C. Licausi F. (2012). Making sense of low oxygen sensing. *Trends Plant Sci.* 17 129–138. 10.1016/j.tplants.2011.12.004 22280796

[B5] Bologa K. L. Fernie A. R. Leisse A. Loureiro M. E. Geigenberger P. (2003). A bypass of sucrose synthase leads to low internal oxygen and impaired metabolic performance in growing potato tubers. *Plant Physiol.* 132 2058–2072. 10.1104/pp.103.022236 12913161 PMC181290

[B6] Bradshaw R. A. Brickey W. W. Walker K. W. (1998). N-Terminal processing: the methionine aminopeptidase and Nα-acetyl transferase families. *Trends Biochem. Sci.* 23 263–267. 10.1016/S0968-0004(98)01227-4 9697417

[B7] Britton M. T. Escobar M. A. Dandekar A. M. (2008). “The oncogenes of *agrobacterium tumefaciens* and agrobacterium rhizogenes,” in *Agrobacterium: From Biology to Biotechnology*, eds Tzfira T. Citovsky V. (New York, NY: Springer), 523–563. 10.1007/978-0-387-72290-0_14

[B8] Bui T. Thompson C. B. (2006). Cancer’s sweet tooth. *Cancer Cell* 9 419–420. 10.1016/j.ccr.2006.05.012 16766260

[B9] Cazzonelli C. I. Velten J. (2003). Construction and testing of an intron-containing luciferase reporter gene fromRenilla reniformis. *Plant Mol. Biol. Report.* 21 271–280. 10.1007/BF02772802

[B10] Chen F. Guo Y. B. Wang J. H. Li J. Y. Wang H. M. (2007). Biological control of grape crown gall by *Rahnella aquatilis* HX2. *Plant Dis.* 91 957–963. 10.1094/PDIS-91-8-0957 30780428

[B11] Chilton M. D. Drummond M. H. Merio D. J. Sciaky D. Montoya A. L. Gordon M. P. (1977). Stable incorporation of plasmid DNA into higher plant cells: the molecular basis of crown gall tumorigenesis. *Cell* 11 263–271. 10.1016/0092-8674(77)90043-5 890735

[B12] De Cleene M. De Ley J. (1976). The host range of crown gall. *Bot. Rev.* 42 389–466. 10.1007/BF02860827

[B13] de Marchi R. Sorel M. Mooney B. Fudal I. Goslin K. Kwaśniewska K. (2016). The N-end rule pathway regulates pathogen responses in plants. *Sci. Rep.* 6:26020. 10.1038/srep26020 27173012 PMC4865862

[B14] Deeken R. Engelmann J. C. Efetova M. Czirjak T. Müller T. Kaiser W. M. (2006). An integrated view of gene expression and solute profiles of arabidopsis tumors: a genome-wide approach. *Plant Cell* 18 3617–3634. 10.1105/tpc.106.044743 17172353 PMC1785400

[B15] Escobar M. A. Dandekar A. M. (2003). *Agrobacterium tumefaciens* as an agent of disease. *Trends Plant Sci.* 8 380–386. 10.1016/S1360-1385(03)00162-6 12927971

[B16] Escobar M. A. Leslie C. A. McGranahan G. H. Dandekar A. M. (2002). Silencing crown gall disease in walnut (*Juglans regia L.*). *Plant Sci.* 163 591–597. 10.1016/S0168-9452(02)00164-4 24083348

[B17] Garzón M. Eifler K. Faust A. Scheel H. Hofmann K. Koncz C. (2007). PRT6/At5g02310 encodes an Arabidopsis ubiquitin ligase of the N-end rule pathway with arginine specificity and is not the CER3 locus. *FEBS Lett.* 581 3189–3196. 10.1016/j.febslet.2007.06.005 17572409

[B18] Gasch P. Fundinger M. Müller J. T. Lee T. Bailey-Serres J. Mustroph A. (2015). Redundant ERF-VII transcription factors bind an evolutionarily-conserved cis-motif to regulate hypoxia-responsive gene expression in arabidopsis. *Plant Cell* 28 160–180. 10.1105/tpc.15.00866 26668304 PMC4746684

[B19] Gelvin S. B. (2003). Agrobacterium-mediated plant transformation: the biology behind the ‘gene-jockeying’ tool. *Microbiol. Mol. Biol. Rev.* 67 16–37. 10.1128/MMBR.67.1.16-37.2003 12626681 PMC150518

[B20] Gibbs D. J. Lee S. C. Isa N. M. Gramuglia S. Fukao T. Bassel G. W. (2011). Homeostatic response to hypoxia is regulated by the N-end rule pathway in plants. *Nature* 479 415–418. 10.1038/nature10534 22020279 PMC3223408

[B21] Gibbs D. J. Md Isa N. Movahedi M. Lozano-Juste J. Mendiondo G. M. Berckhan S. (2014). Nitric oxide sensing in plants is mediated by proteolytic control of group VII ERF transcription factors. *Mol. Cell* 53 369–379. 10.1016/j.molcel.2013.12.020 24462115 PMC3969242

[B22] Giuntoli B. Lee S. C. Licausi F. Kosmacz M. Oosumi T. van Dongen J. T. (2014). A trihelix DNA binding protein counterbalances hypoxia-responsive transcriptional activation in arabidopsis. *PLoS Biol.* 12:e1001950. 10.1371/journal.pbio.1001950 25226037 PMC4165759

[B23] Gogvadze V. Orrenius S. Zhivotovsky B. (2008). Mitochondria in cancer cells: what is so special about them? *Trends Cell Biol.* 18 165–173. 10.1016/J.TCB.2008.01.006 18296052

[B24] Gohlke J. Deeken R. (2014). Plant responses to *Agrobacterium tumefaciens* and crown gall development. *Front. Plant Sci.* 5:155. 10.3389/fpls.2014.00155 24795740 PMC4006022

[B25] Gravot A. Richard G. Lime T. Lemarié S. Jubault M. Lariagon C. (2016). Hypoxia response in arabidopsis roots infected by Plasmodiophora brassicae supports the development of clubroot. *BMC Plant Biol.* 16:251. 10.1186/s12870-016-0941-y 27835985 PMC5106811

[B26] Guyon P. Chilton M. D. Petit A. Tempé J. (1980). Agropine in ‘null-type’ crown gall tumors: evidence for generality of the opine concept. *Proc. Natl. Acad. Sci. U.S.A.* 77 2693–2697. 10.1073/pnas.77.5.2693 16592823 PMC349469

[B27] Holman T. J. Jones P. D. Russell L. Medhurst A. Ubeda Tomás S. Talloji P. (2009). The N-end rule pathway promotes seed germination and establishment through removal of ABA sensitivity in arabidopsis. *Proc. Natl. Acad. Sci. U.S.A.* 106 4549–4554. 10.1073/pnas.0810280106 19255443 PMC2649959

[B28] Igamberdiev A. U. Baron K. Manac’h-Little N. Stoimenova M. Hill R. D. (2005). The haemoglobin/nitric oxide cycle: involvement in flooding stress and effects on hormone signalling. *Ann. Bot.* 96 557–564. 10.1093/aob/mci210 16027133 PMC4247025

[B29] Ismond K. P. Dolferus R. de Pauw M. Dennis E. S. Good A. G. (2003). Enhanced low oxygen survival in arabidopsis through increased metabolic flux in the fermentative pathway. *Plant Physiol.* 132 1292–1302. 10.1104/PP.103.022244 12857811 PMC167069

[B30] Jefferson R. A. Kavanagh T. A. Bevan M. W. (1987). GUS fusions: beta-glucuronidase as a sensitive and versatile gene fusion marker in higher plants. *EMBO J.* 6 3901–3907. 10.1002/j.1460-2075.1987.tb02730.x 3327686 PMC553867

[B31] Kim J. Dang C. V. (2006). Cancer’s molecular sweet tooth and the warburg effect. *Cancer Res.* 66 8927–8930. 10.1158/0008-5472.CAN-06-1501 16982728

[B32] Klinkenberg J. Faist H. Saupe S. Lambertz S. Krischke M. Stingl N. (2014). Two fatty acid desaturases, stearoyl-acyl carrier protein Δ9-desaturase6 and fatty acid desaturase3, are involved in drought and hypoxia stress signaling in arabidopsis crown galls. *Plant Physiol.* 164 570–583. 10.1104/pp.113.230326 24368335 PMC3912090

[B33] Kosmacz M. Parlanti S. Schwarzländer M. Kragler F. Licausi F. Van dongen J. T. (2015). The stability and nuclear localization of the transcription factor RAP2.12 are dynamically regulated by oxygen concentration. *Plant Cell Environ.* 38 1094–1103. 10.1111/pce.12493 25438831

[B34] Licausi F. Kosmacz M. Weits D. A. Giuntoli B. Giorgi F. M. Voesenek L. A. C. J. (2011). Oxygen sensing in plants is mediated by an N-end rule pathway for protein destabilization. *Nature* 479 419–422. 10.1038/nature10536 22020282

[B35] Livak K. J. Schmittgen T. D. (2001). Analysis of relative gene expression data using real-time quantitative PCR and the 2^−ΔΔC_T_^ method. *Methods* 25 402–408. 10.1006/meth.2001.1262 11846609

[B36] Lopez-Lazaro M. (2008). The warburg effect: why and how do cancer cells activate glycolysis in the presence of oxygen? *Anticancer. Agents Med. Chem.* 8 305–312. 10.2174/187152008783961932 18393789

[B37] Melnyk C. W. (2017). Connecting the plant vasculature to friend or foe. *New Phytol.* 213 1611–1617. 10.1111/nph.14218 27716935

[B38] Mustroph A. Lee S. C. Oosumi T. Zanetti M. E. Yang H. Ma K. (2010). Cross-kingdom comparison of transcriptomic adjustments to low-oxygen stress highlights conserved and plant-specific responses. *Plant physiol.* 152 1484–1500. 10.1104/pp.109.151845 20097791 PMC2832244

[B39] Păcurar D. I. Thordal-Christensen H. Păcurar M. L. Pamfil D. Botez C. Bellini C. (2011). *Agrobacterium tumefaciens*: from crown gall tumors to genetic transformation. *Physiol. Mol. Plant Pathol.* 76 76–81. 10.1016/J.PMPP.2011.06.004

[B40] Pfeiffer T. Schuster S. Bonhoeffer S. (2001). Cooperation and competition in the evolution of atp-producing pathways. *Science* 292 504–507. 10.1126/science.1058079 11283355

[B41] Riber W. Müller J. T. Visser E. J. W. Sasidharan R. Voesenek L. A. C. J. Mustroph A. (2015). The greening after extended darkness1 is an N-end rule pathway mutant with high tolerance to submergence and starvation. *Plant Physiol.* 167 1616–1629. 10.1104/pp.114.253088 25667318 PMC4378152

[B42] Schito L. Semenza G. L. (2016). Hypoxia-inducible factors: master regulators of cancer progression. *Trends Cancer* 2 758–770. 10.1016/j.trecan.2016.10.016 28741521

[B43] Semenza G. L. (2012). Hypoxia-inducible factors in physiology and medicine. *Cell* 148 399–408. 10.1016/J.CELL.2012.01.021 22304911 PMC3437543

[B44] Spitzer M. Wildenhain J. Rappsilber J. Tyers M. (2014). BoxPlotR: a web tool for generation of box plots. *Nat. Methods* 11 121–122. 10.1038/nmeth.2811 24481215 PMC3930876

[B45] Sugimoto K. Jiao Y. Meyerowitz E. M. (2010). Arabidopsis regeneration from multiple tissues occurs via a root development pathway. *Dev. Cell* 18 463–471. 10.1016/J.DEVCEL.2010.02.004 20230752

[B46] Tasaki T. Sriram S. M. Park K. S. Kwon Y. T. (2012). The N-end rule pathway. *Annu. Rev. Biochem.* 81 261–289. 10.1146/annurev-biochem-051710-093308 22524314 PMC3610525

[B47] Thomashow M. F. Panagopoulos C. G. Gordon M. P. Nester E. W. (1980). Host range of *Agrobacterium tumefaciens* is determined by the Ti plasmid. *Nature* 283 794–796. 10.1038/283794a0

[B48] Tzfira T. Citovsky V. (2006). Agrobacterium-mediated genetic transformation of plants: biology and biotechnology. *Curr. Opin. Biotechnol.* 17 147–154. 10.1016/j.copbio.2006.01.009 16459071

[B49] Varshavsky A. (2011). The N-end rule pathway and regulation by proteolysis. *Protein Sci.* 20 1298–1345. 10.1002/pro.666 21633985 PMC3189519

[B50] Vaupel P. Harrison L. (2004). Tumor hypoxia: causative factors, compensatory mechanisms, and cellular response. *Oncologist* 9 4–9. 10.1634/theoncologist.9-90005-4 15591417

[B51] Vicente J. Mendiondo G. M. Pauwels J. Pastor V. Izquierdo Y. Naumann C. (2018). Distinct branches of the N-end rule pathway modulate the plant immune response. *New Phytol.* 221 988–1000. 10.1111/nph.15387 30117535

[B52] Weits D. A. Giuntoli B. Kosmacz M. Parlanti S. Hubberten H.-M. Riegler H. (2014). Plant cysteine oxidases control the oxygen-dependent branch of the N-end-rule pathway. *Nat. Commun.* 5:3425. 10.1038/ncomms4425 24599061 PMC3959200

[B53] White M. D. Kamps J. J. A. G. East S. Taylor Kearney L. J. Flashman E. (2018). The plant cysteine oxidases from *Arabidopsis thaliana* are kinetically tailored to act as oxygen sensors. *J. Biol. Chem.* 293 11786–11795. 10.1074/jbc.RA118.003496 29848548 PMC6066304

[B54] White M. D. Klecker M. Hopkinson R. J. Weits D. A. Mueller C. Naumann C. (2017). Plant cysteine oxidases are dioxygenases that directly enable arginyl transferase-catalysed arginylation of N-end rule targets. *Nat. Commun.* 8:14690. 10.1038/ncomms14690 28332493 PMC5376641

[B55] Wu H.-Y. Liu K.-H. Wang Y.-C. Wu J.-F. Chiu W.-L. Chen C.-Y. (2014). AGROBEST: an efficient Agrobacterium-mediated transient expression method for versatile gene function analyses in Arabidopsis seedlings. *Plant Methods* 10:19. 10.1186/1746-4811-10-19 24987449 PMC4076510

[B56] Young J. M. Kuykendall L. D. Martínez-Romero E. Kerr A. Sawada H. (2001). A revision of Rhizobium Frank 1889, with an emended description of the genus, and the inclusion of all species of Agrobacterium Conn 1942 and Allorhizobium undicola de Lajudie et al. 1998 as new combinations: rhizobium radiobacter, R. rhizogenes, R. rubi, R. undicola and R. vitis. *Int. J. Syst. Evol. Microbiol.* 51 89–103. 10.1099/00207713-51-1-89 11211278

[B57] Zeng Y. Wu Y. Avigne W. T. Koch K. E. (1999). Rapid repression of maize invertases by low oxygen. invertase/sucrose synthase balance, sugar signaling potential, and seedling survival. *Plant Physiol.* 121 599–608. 10.1104/pp.121.2.599 10517852 PMC59423

[B58] Zipfel C. Kunze G. Chinchilla D. Caniard A. Jones J. D. G. Boller T. (2006). Perception of the bacterial PAMP EF-Tu by the receptor EFR restricts agrobacterium-mediated transformation. *Cell* 125 749–760. 10.1016/j.cell.2006.03.037 16713565

