# Supplementary material for: Hypoxic Conditions in Crown Galls Induce Plant Anaerobic Responses That Support Tumor Proliferation
Source: Front Plant Sci. 2019 Feb 5;10:56. doi: 10.3389/fpls.2019.00056 (PMC6371838; doi:10.3389/fpls.2019.00056)
Supplement: Supplementary file 1 [file Data_Sheet_1.docx]

**Supplemental Table S1.** Full list of the vectors used with their references

| **Final construct** | **Original vector** | **Reference** |
| --- | --- | --- |
| promPCO1:GFP-GUS | pKGWFS7 | (Karimi et al., 2002) |
| promHRPEX5:GFP-GUS | pKGWFS7 | (Karimi et al., 2002) |
|  |  |  |

**Supplementary Table S2.** Full list of the primers used in this study. (F = forward primer; R = reverse primer)

| **Primer name:** | **5'-3' sequence** |
| --- | --- |
| gwPPCO1fw | CACCAAAATTTCTTTTAATTTACTATTGATG |
| gwPPCO1rv | TCTCTGGTTTCATCTCAAACCCCAT |
| sgAt5G15120_F | ATTGGGTGGTTGATGCTCCAATG |
| sgAt5g15120_R | ATGCATGTTCCCGCCATCTTC |
| sgAt4g05320 F | GGCCTTGTATAATCCCTGATGAATAAG |
| sgAt4g05320 R | AAAGAGATAACAGGAACGGAAACATAGT |
| sgAt4g33070 F | CACAGAATCTTCAATGTTCTTACC |
| sgAt4g33070 R | CCATGATAAAGCGTACATGGAA |
| sgAt3g10040 F | ACAACCACCGCAACAGAATCC |
| sgAt3g10040 R | TCTCCGCAATTCTCGCCAT |
| sgAt5g39890_F | CTTCGAGCCGTTTTGGATGA |
| sgAt5g39890_R | ACGTCACTAACGGAGATCGTCC |
| sgAt1g77120 F | TATTCGATGCAAAGCTGCTGTG |
| sgAt1g77120 R | CGAACTTCGTGTTTCTGCGGT |
| sgAt2g16060_F | TTTGAGGTGGCCAAGTATGCA |
| sgAt2g16060_R | TGATCATAAGCCTGACCCCAA |
| sgAt5g20830_F | ACGCTGAACGTATGATAACGCG |
| sgAt5g20830_R | AACCCTGGAAAGCAAGGCAAG |
| sgAt3g43190_F | CGCAGAACGTGTAATAACGCG |
| sgAt3g43190_R | CAACCCTTGAGAGCAAAGCAAA |
| sgAT3G02550_F | TGAAGCGCAAGCTAACGCA |
| sgAT3G02550_R | ATCCCAGGACGAAGGTGATTG |
| sgAT1G43800_F | TTGGCAACCCGCTTCTTTCTTACCD |
| sgAT1G43800_R | TTTCCCTCAGCTCACGAACCTG |

**Supplemental Table S3.** Comparison of the mRNA levels of hypoxia-inducible genes in crown galls, or uninfected stem tissue as control. Data are represented as means relative to the uninfected stem tissue ± s.d. for n=4. *P*-values calculated using one-way ANOVA and Holm-Sidak post-hoc test)

|  | **Stem tissue** | | **Crown gall** | |  |
| --- | --- | --- | --- | --- | --- |
|  | **Fold change** | | **Fold change** | |  |
| **Gene** | **(log2)** | **st.Dev** | **(log2)** | **st.Dev** | **P-val** |
| ***ADH1*** | 0.00 | 0.617 | 2.963 | 0.989 | 0.05 |
| ***PDC1*** | 0.00 | 0.623 | 6.188 | 1.108 | 0.03 |
| ***HB1*** | 0.00 | 0.996 | 4.100 | 0.473 | < 0.01 |
| ***SUS1*** | 0.00 | 0.353 | 3.861 | 0.852 | < 0.01 |
| ***SUS4*** | 0.00 | 0.859 | 6.981 | 0.706 | < 0.01 |
| ***PCO1*** | 0.00 | 0.403 | 2.268 | 0.142 | < 0.01 |
| ***PCO2*** | 0.00 | 0.541 | 4.147 | 0.496 | < 0.01 |
| ***HRA1*** | 0.00 | 0.600 | 4.479 | 0.734 | < 0.01 |
| ***LBD41*** | 0.00 | 1.317 | 4.292 | 0.540 | < 0.01 |
| ***SAD6*** | 0.00 | 1.907 | 9.433 | 0.651 | < 0.01 |
